# Extensive antibody search with whole spectrum black-box optimization

**DOI:** 10.1038/s41598-023-51095-z

**Published:** 2024-01-04

**Authors:** Andrejs Tučs, Tomoyuki Ito, Yoichi Kurumida, Sakiya Kawada, Hikaru Nakazawa, Yutaka Saito, Mitsuo Umetsu, Koji Tsuda

**Affiliations:** 1 Graduate School of Frontier Sciences, The University of Tokyo, Kashiwa, Japan; 2 Department of Biomolecular Engineering, Graduate School of Engineering, Tohoku University, Sendai, Japan; 3 Artificial Intelligence Research Center, National Institute of Advanced Industrial Science and Technology (AIST), Tokyo, Japan; 4 RIKEN Center for Advanced Intelligence Project, RIKEN, Tokyo, 103-0027 Japan; 5 AIST-Waseda University Computational Bio Big-Data Open Innovation Laboratory (CBBD-OIL), Tokyo, Japan; 6 Center for Basic Research on Materials, National Institute for Materials Science (NIMS), Tsukuba, Japan; 7 Department of Data Science, School of Frontier Engineering, Kitasato University, Sagamihara, Japan

**Keywords:** Machine learning, Protein design

## Abstract

In designing functional biological sequences with machine learning, the activity predictor tends to be inaccurate due to shortage of data. Top ranked sequences are thus unlikely to contain effective ones. This paper proposes to take prediction stability into account to provide domain experts with a reasonable list of sequences to choose from. In our approach, multiple prediction models are trained by subsampling the training set and the multi-objective optimization problem, where one objective is the average activity and the other is the standard deviation, is solved. The Pareto front represents a list of sequences with the whole spectrum of activity and stability. Using this method, we designed VHH (Variable domain of Heavy chain of Heavy chain) antibodies based on the dataset obtained from deep mutational screening. To solve multi-objective optimization, we employed our sequence design software MOQA that uses quantum annealing. By applying several selection criteria to 19,778 designed sequences, five sequences were selected for wet-lab validation. One sequence, 16 mutations away from the closest training sequence, was successfully expressed and found to possess desired binding specificity. Our whole spectrum approach provides a balanced way of dealing with the prediction uncertainty, and can possibly be applied to extensive search of functional sequences.

## Introduction

Machine learning-based protein design strategies can be classified into two categories according to how the training dataset is collected: computational^1,2^ and experimental^3–5^. In the former category, protein structures of interest are predicted by a structure predictor such as AlphaFold2^6^ or RoseTTafold^7^. The designed proteins are computationally evaluated on the basis of closeness to desired topology^2^ or simulated binding affinities to target proteins^1^. In the latter category, the dataset is made by synthesizing as many protein variants as possible and evaluated by experimental means.^4^ To this aim, deep mutational screening^8^ is often used in combination with random mutagenesis. However, the number of sequences available for training is far smaller than computational data collection^3^.

Prediction uncertainty is a central topic in automatic design with small data^9^. The prediction at a point in a sample abundant region is more certain than that in a sample scarce region. Bayesian optimization^10^, a popular automatic design algorithm, treats uncertainty as *opportunity*, because its recommendation scores such as expected improvement increase as uncertainty increases. LaMBO^11^ and guided diffusion models^12^ belong to this type of adventurous approach. In contrast, the methods based on the domain of applicability^13^ (DoA) restrict recommendation within sample abundant regions, regarding uncertainty as *risk*. Given an automatic design task at hand, it is difficult to choose one from these two approaches, because the outcome depends on the accuracy of predicted scores and their uncertainty estimates.

**Figure 1:**
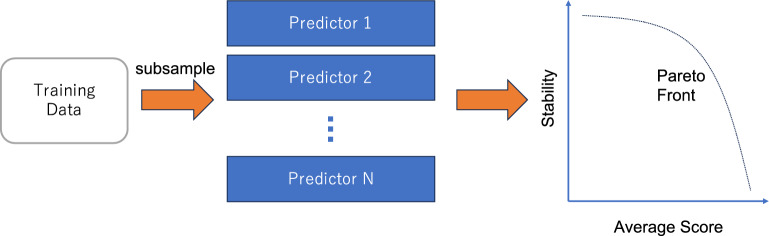
Whole spectrum black-box optimization. Multiple datasets are created by subsampling and used to train an ensemble of predictors. A multi-objective optimization problem is defined such that the average of predicted scores and their stability are maximized.

In our approach, we consider the whole spectrum of adventurous and conservative design strategies and try all of them collectively based on the framework of multi-objective optimization^14^ (Fig. 1). First, variations of the training datasets are created by subsampling. A machine learning model is trained by the multiple training datasets, yielding an ensemble of predictors. A multi-objective optimization problem^14^ is postulated such that an objective function is the average of all prediction scores and the other is their standard deviation (i.e., prediction instability). By applying a solver^14^, one can identify several solutions at the Pareto front, i.e., the set of non-dominated solutions where no objective function can be improved without sacrificing the other at each solution. These solutions range from high average, low stability ones to low average, high stability ones. In biological sequence design problems, the solutions correspond to candidate sequences for wet-lab validation. Since only a few sequences can be validated, domain experts are asked to select some of them. Our approach can produce a diverse list of sequences in comparison to the naïve approach where only one predictor is used and the sequences are ranked by its prediction score. A practical advantage of our approach is that additional objective functions such as developability measures can easily be incorporated to the multi-objective problem.

We apply our approach to discover VHH (Variable domain of Heavy chain of Heavy chain) antibodies against galectin-3, which is a potential therapeutic target for cancer treatment and diagnostic biomarkers for several diseases, including heart failure and cancers^15^. 12,737 labeled sequences are obtained by deep mutational screening and five predictors are trained by subsampling the dataset. We applied our multi-objective optimization software MOQA^16^ to optimize the following three objectives: (1) the average of the prediction scores, (2) the standard deviation of them, (3) solubility predicted by NetSolP^17^. Notice that MOQA is based on quantum annealing^18^ and deep learning^19^, and was previously applied to antimicrobial peptide design^16^. See Supplementary Information about the algorithm of MOQA. NetSolP solubility score was chosen due to high accuracy enabled by protein language models^17^. From 19,778 generated sequences, five were chosen based on our quality and novelty criteria. The five were synthesized and evaluated in wet lab experiments, and one sequence, which is distinctly different from the training sequences, was found to possess desired binding affinity and specificity. This result implies that our method called WS-MOQA enables far-reaching search of functional sequences.

## Results

### Data preparation

**Figure 2:**
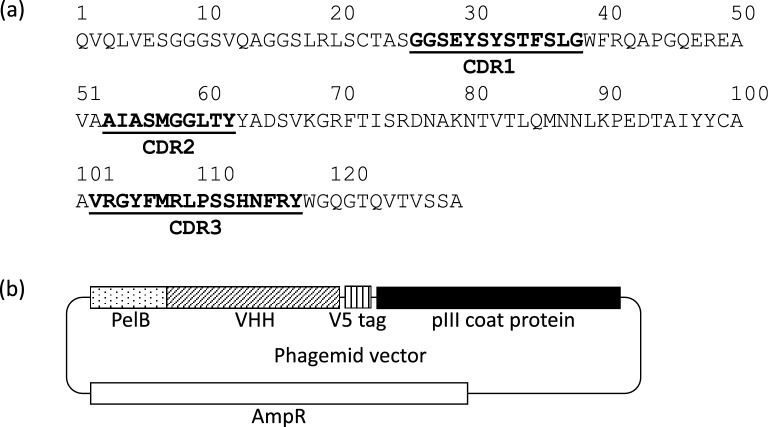
DNA sequence of VHH in the phagemid vector. (**a**) The amino acid sequence of wild-type VHH (3DWT). Each CDR was shown in bold and underlined. (**b**) The phagemid vector containing the coding region for VHH.

To collect the training set, we performed four rounds of phage display biopanning^20^ to select VHH sequences bound to galectin-3. For the phage display library, the complementarity determining regions of CDR1 (13 aa), CDR2 (10 aa), and CDR3 (16 aa) in VHH (PDB ID: 3DWT) were randomized with degenerate codons designed to mimic an amino acid frequency of antibody CDRs^21^, and the variants were displayed on phages (Fig. 2). The prepared initial phage library (library size: 8.6 × 10^7^) was used to perform biopanning against galectin-3. In the biopanning, the following steps were iterated four times: (1) removal of non-specifically bound phages (N.S. phages), (2) interaction with antigen and elution of target-binding phages (eluted phages), (3) infection of the selected phage into *E. coli*, and 4) amplification of phages (Fig S1). The N.S. phages and eluted phages in the fourth round, were subjected to deep sequencing analysis with the MiSeq platform. Raw sequences were filtered and trimmed according to their quality, and the forward reads were merged with the corresponding reverse reads. As a result, we obtained 178,883 reads in N.S. phages and 133,179 reads in eluted phages. As the training set, we retained sequences of more than three reads of sequence in either N.S. phages or eluted phages (12,737 sequences). The binding score of each sequence is defined as

$$ Score = \frac{frequency\;in\;eluted\;phages\;at\;the\;4th\;round}{{frequency\;in\;N.S.\;phages\;at\;the\;4th\;round}} $$

Sequences with Score ≥ 5 were considered to bind (i.e., positive class), while sequences with Score < 1 were unbound (i.e., negative class).

### Whole spectrum black-box optimization

**Figure 3:**
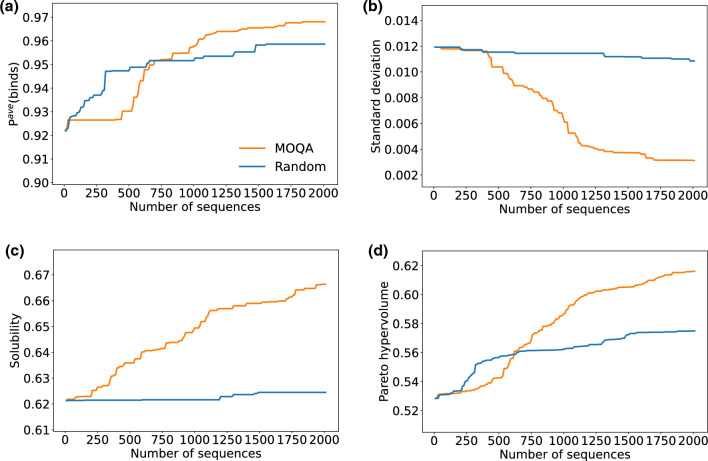
Multi-objective optimization. Development of sequence optimization as sequences are generated. Each curve corresponds to the value of the best sequence generated so far. The results of random generation are shown for reference. Each panel corresponds to the following performance measures: (**a**) average prediction score; (**b**) standard deviation of prediction scores; (**c**) solubility; (**d**) pareto hypervolume.

From the training set, five datasets are created by first splitting the negative sequences into five equal-sized subsets and combining each of them with the positive sequences. Each dataset was used to train bidirectional LSTM using PARROT python package^22^. This model outputs the probability of the input sequence being in the positive class, which is used as our prediction score. The model had one hidden layer with hidden vector size 10. It was trained for 25 epochs with the learning rate set 0.001. One-hot encoding was used for sequence representation. Our multi-objective optimization problem is concerned about the prediction score average, the standard deviation and solubility predicted by NetSolP^17^. 20,000 VHH sequences were generated by MOQA in ten independent runs (each with different random initialization). Figure 3 shows the development of the three objective functions and their Pareto hypervolume^23^. It is found that all functions are optimized close to saturation. After removing duplicates, 19,778 sequences remained. See Fig. S2 for tSNE visualization of the training sequences and generated sequences.

For wet-lab validation, we selected the sequences satisfying the following constraints. (1) The sequence length is 39, that is equal to the combined length of CDRs; (2) it does not contain repetitive amino acids of length 5 or more; (3) the average score is above 0.6; (4) solubility scores from CamSol^24^ are above 0.0; (5) the CDR1, CDR2 and CDR3 sequences differ from the wild-type by at least three residues. As a result, the five sequences shown in Table 1 were selected. Notably, all of them were distinctly different from the training sequences. The Hamming distance from each sequence to its closest training sequence was between 16 and 23.

**Table 1:** Five VHHs selected for wet-lab validation. The Hamming distance from the closest training sequence is shown as well.

Name	CDR1	CDR2	CDR3	Distance
WT	GGSEYSYSTFSLG	AIASMGGLTY	VRGYFMRLPSSHNFRY	–
VHH1832	SNQNNSNYNSYDY	YFYNNSSYSY	DSFNYYDDNNFDDYRR	23
VHH1834	ANGDNYNASYSDN	YNYCDYSYSY	DDYFNYYYNNNDDDRF	23
VHH1835	DNQNLNSYNDNNS	FSYNSYYGSY	DDYYYSYYNNFDNDRA	22
VHH1836	NNNDNSNNYSYYD	SFYGDFNFYY	NNQPNDDHYDVYDHNS	16
VHH1837	NQANFNSHRSYSS	NNGSYYDDSF	VNPPNDDHYDVYDHNS	18

### Validation of selected VHHs

**Figure 4:**
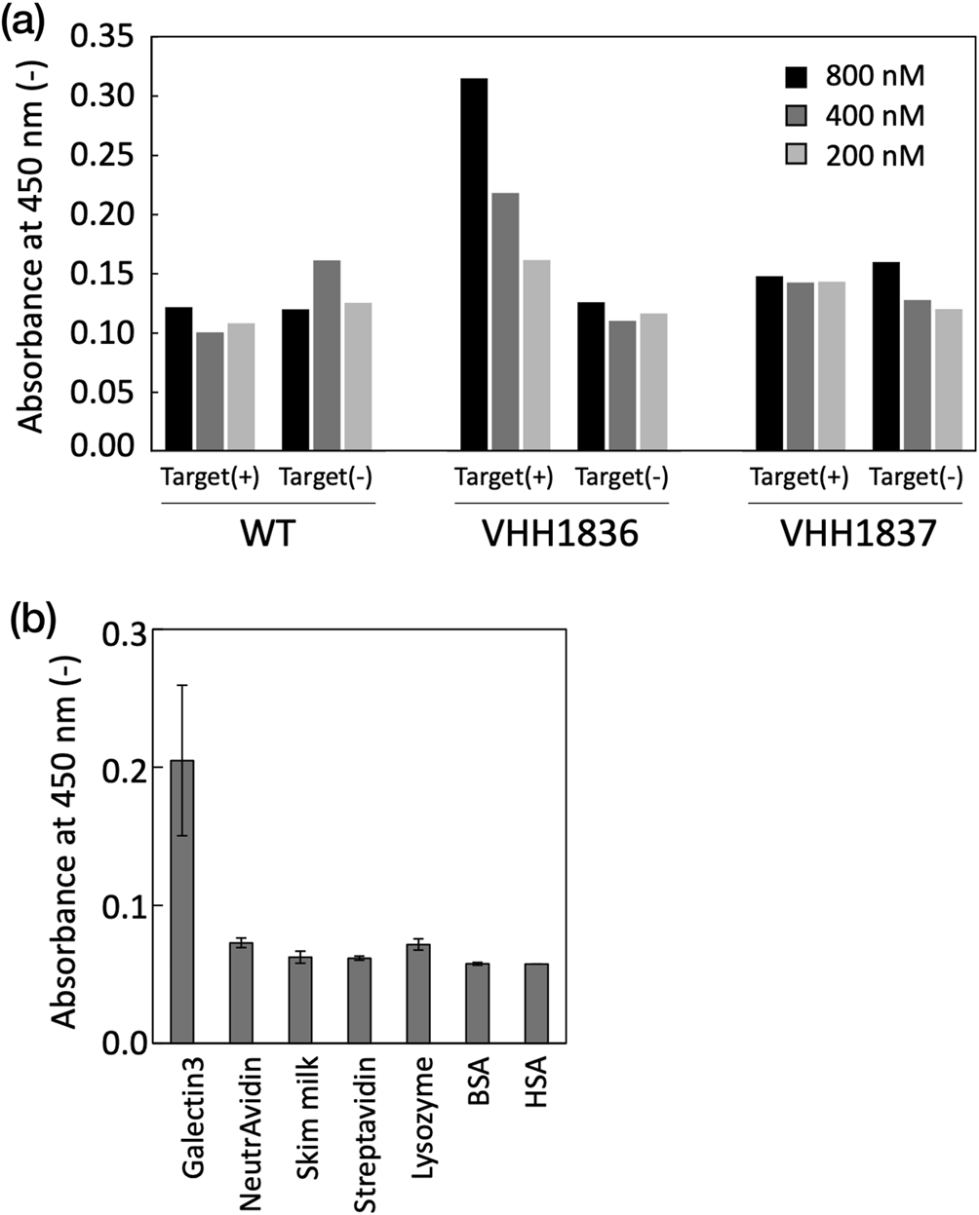
ELISA. The binding function of proposed VHHs: (**a**) ELISA of purified wild-type 3DWT, VHH1836, and VHH1837 at the concentration of 800 nM (black), 400 nM (gray), and 200 nM (light gray). ELISA was measured for the plates with and without galectin-3 [Target ( +), Target(−), respectively]. (**b**) ELISA of VHH1836 for galectin-3, NeutrAvidin, skim milk, streptavidin, lysozyme from chicken egg, bovine serum albumin, and human serum albumin. The concentration of VHH1836 was at 800 nM. Error bars indicate the standard deviation for three trials.

The expression vectors bearing the gene of the five VHH variants (VHH1832, VHH1834, VHH1835, VHH1836, VHH1837) were prepared and *E. coli* BL21(DE3) cells were transformed with the vectors. The five variants generated in *E. coli* were purified by means of immobilized metal ion affinity chromatography (IMAC) and size exclusion chromatography (SEC). The expression levels of the WT, VHH1836, and VHH1837 were 11 mg/L-broth, 6 × 10^–2^ mg/L-broth, and 8 × 10^–3^ mg/L-broth, respectively. The designed sequences yielded lower expression than WT, but they nevertheless formed monomers (Fig. S3). The fractions of monomers were collected and used to measure the binding affinity to galectin-3 by means of enzyme-linked immunosorbent assay (ELISA). As a result, VHH1836 bound specifically to the plates where galectin-3 was immobilized, but not to the plates without galectin-3 (Fig. 4a). VHH1836 showed concentration-dependent binding, while WT did not bind to the galectin-3 at the same concentration range (800 nM, 400 nM, and 200 nM of VHHs). In addition, VHH1836 showed little binding to other proteins such as streptavidin, lysozyme from chicken egg, bovine serum albumin, and human serum albumin (Fig. 4b). The CD spectrum of VHH1836 shows that the VHH forms a beta-rich secondary structure like immunoglobulin-fold (Fig. S4). The CD spectra of VHH1836 differed largely from those of WT probably due to the structural change of the CDR loop structure.

## Discussion

Our methodological contribution is the proposal of including stability as an additional objective function. In our paper, we used MOQA but our idea can be applied to any multi-objective optimization solver, such as CMA-ES ^14^, LaMBO^11^ and guided diffusion models^12^. Formulating the stability-activity balancing as multi-objective optimization, one does not need to manually set the balancing constant as done by Tran et al.^25^. Covering the Pareto front with solutions helps to improve diversity, but it may not be realistic when the number of objective functions is more than three. Our approach is reminiscent of bagging^26^, where multiple prediction models are created by subsampling the training dataset and the average of prediction scores are used for making decisions for new examples. However, our approach aims to improve black-box optimization, while the prediction accuracy is the primary concern in bagging. We used random splitting to derive the stability, but other sampling methods such as bootstrapping or sampling with replacement may be used.

Our approach, whole spectrum black-box optimization, creates diverse solutions that balance activity and stability. It was successfully applied to the task of finding innovative antibodies that are distinctly different from training sequences. Our results suggest that it has the potential to be applied to a wide range of biological, chemical, and pharmaceutical design problems.

## Materials and method

### Library construction

The biopanning was conducted basically according to previous report^4,5^. CDR1, CDR2, and CDR3 in 3DWT were randomized using degenerate codons reflecting an amino acid frequency of antibody CDRs for training data. M13 phage libraries displaying VHH variants with a size of 8.6 × 10^7^ were prepared. Colony-forming units (5.0 × 10^11^) from an M13 phage library displaying VHH variants were exposed to magnetic beads (Dynabeads MyOne Streptavidin T1; Thermo Fisher Scientific, MA, USA) for 60 min at room temperature (negative selection in Fig S2) and centrifuged to separate supernatant and magnetic beads. The phages bound to the beads were collected as N.S. phages. For target preparation, 2 µM galectin-3 in PBS was incubated with magnetic beads for 30 min at 4 °C. The supernatant was incubated with galectin-3–immobilized magnetic beads for 60 min at room temperature, and the beads were washed 10 times with PBS with 0.05% Tween-20 for 5 min each wash. Bound phages were eluted with 100 µL of triethylamine and neutralized with 300 µL of 0.5 M Tris-HCl (pH 6.8). Log-phase *E. coli* TG-1 cells were incubated overnight at 37 °C with 200 µL of the eluted phages in 2× YT agar medium containing 100 µg/mL ampicillin and 1% (w/v) glucose. Cells grown on the plates were used to prepare phage particles for the next round.

### Sample preparation and deep sequencing

After fourth round of biopanning, polyclonal plasmid DNAs were extracted with phenol–chloroform from N.S. phages and eluted phages. The extracted DNAs were used for the first polymerase chain reaction (PCR) to amplify VHH library fragments with the primers containing an annealing region for the second PCR primers. The PCR products were purified by using 1.5% agarose gel and a Qiaex II Gel Extraction Kit (20051; Qiagen, Hilden, Germany) and subjected to the second PCR to attach adapter sequences containing TruSeq DNA CD Indexes. The resulting fragments were purified as above, quantified by using a Qbit™ 1× dsDNA HS Assay Kit (Q33231; Thermo Fisher Scientific), and pooled in equal amounts. The quality of the libraries was checked by using an Agilent 2100 Bioanalyzer (G2939B; Agilent Technologies, CA, USA). The prepared sample was sequenced on the MiSeq platform (Illumina, CA, USA) by using a MiSeq Reagent Kit v3 (15043895; Illumina) with 2 × 300 bp paired end reads.

### Preparation of proposed VHHs and galectin-3

The gene of VHHs were each inserted into the *Nco* I–*Sac* II site of the pRA vector, which included FLAG and poly-histidine tags^27^. *E. coli* BL21(DE3) cells were transformed with the constructed expression vectors, grown overnight at 28 °C on LB agar, and then cultured in 2× YT broth; both media contained 100 µg/mL ampicillin. Isopropyl-β-d-thiogalactopyranoside (IPTG) was added to a final concentration of 1 mM at OD_600_ = 0.8, and the cells were shaken at 160 rpm for overnight at 28 °C. IPTG was added to the flask to a final concentration of 1 mM. The cells were shaken at 160 rpm at 20 °C overnight. The cells were harvested by centrifugation, resuspended in phosphate-buffered saline (PBS), and sonicated. Insoluble matter was removed by centrifugation. Variants were purified from the supernatants by IMAC (Ni Sepharose™ 6 Fast Flow; Cytiva, IL, USA) and SEC (HiLoad 26/600 Superdex 75 pg; Cytiva, IL, USA). The procedure of the preparation of galectin-3 was described previously^4^.

### Enzyme-linked immunosorbent assay (ELISA)

Fifty microliters of 4 µg/mL NeutrAvidin (Thermo Fisher Scientific) in PBS were incubated in the wells of a 96-well polystyrene enzyme-linked immunosorbent assay microplate (655061; Greiner, Austria) for 60 min, then 150 µL of 3% (w/v) skim milk in PBS was added to the wells, and the plates were incubated for a further 30 min for blocking. After washing each well with PBS three times, 50 µL of 10 µg/mL biotinylated galectin-3 solution was added and the wells were incubated for 30 min. The wells were washed again with PBS, and VHH solution was added and the wells were incubated for 30 min. After washing each well with PBS/0.05% Tween20 three times, the wells were incubated for 40 min at room temperature with horseradish peroxidase-conjugated mouse anti-FLAG monoclonal antibody (1:10,000; A8592, Sigma Aldrich). After washing each well with PBS/0.05% Tween20 three times, 50 µL of 3,3′,5,5′-tetramethylbenzidine solution (1-step Ultra TMB-ELISA Substrate Solution; Thermo Fisher Scientific) was added and the wells were incubated for 10 min at room temperature. After incubation, 50 µL of 2 M H_2_SO_4_ was added to each well and absorbance at 450 nm was measured with a Synergy H4 Hybrid Multimode Microplate Reader (BioTek Japan, Tokyo, Japan). In the case of measuring target specificity, 50 µL of 4 µg/mL streptavidin, lysozyme, bovine serum albumin, and human serum albumin were incubated in the wells of a 96-well polystyrene ELISA microplate before skim milk blocking and addition of VHHs.

### Circular dichroism (CD) spectra

CD spectra were measured with a J-820 CD spectrometer (Jasco, Japan) in a 1.0-mm-long quartz cuvette, as follows: band width 1.0 nm, resolution 0.1 nm, response 8 s, scan speed 2 nm/min. The concentrations of purified VHHs variants were 5 µM.

### Supplementary Information

Supplementary Information.

## Data Availability

The code and datasets are available at Github repository, https://github.com/tucs7/nanobody_MOQA.
