# Supplementary material for: Extensive antibody search with whole spectrum black-box optimization
Source: Sci Rep. 2024 Jan 4;14:552. doi: 10.1038/s41598-023-51095-z (PMC10767033; doi:10.1038/s41598-023-51095-z)
Supplement: Supplementary file 1 — Supplementary Information. [file 41598_2023_51095_MOESM1_ESM.pdf]

Supplementary Information: Extensive Antibody Search with
Whole Spectrum Black-box Optimization

Andrejs Tučs[1#], Tomoyuki Ito[2#], Yoichi Kurumida[3,7], Sakiya Kawada[2], Hikaru Nakazawa[2], Yutaka Saito[1,3,4,5,7], Mitsuo Umetsu[2,4*], Koji Tsuda[1,4,6*]

[1] Graduate School of Frontier Sciences, The University of Tokyo, Kashiwa, Japan. [2]Department of Biomolecular Engineering, Graduate School of Engineering, Tohoku University, Sendai, Japan. [3]Artificial Intelligence Research Center, National Institute of Advanced Industrial Science and Technology (AIST), Tokyo, Japan. [4]RIKEN Center for Advanced Intelligence Project, RIKEN, Tokyo, 103-0027 Japan. [5]AIST-Waseda University Computational Bio Big-Data Open Innovation Laboratory (CBBD-OIL), Tokyo, Japan. [6]Center for Basic Research on Materials, National Institute for Materials Science (NIMS), Tsukuba, Japan. [7]Department of Data Science, School of Frontier Engineering, Kitasato University, Sagamihara, Japan.

#These authors contributed equally.

MOQA

MOQA uses binary variational autoencoder to convert sequences to binary bit vector. We summarize multiple properties into one score y based on the distance from the current pareto front and construct a predictor of the score in the following quadratic form,

$$y = \sum_{i=1}^{M} h_i x_i + \sum_{i,j=1}^{M} J_{ij} x_i x_j$$

where x_i is an element of the binary vector. The parameters h_i, J_{ij} are obtained by training a factorization machine (FM) from the pairs of the binary vectors and associated scores. The functional form of FM is the following

$$y = \sum_{i=1}^{M} h_i\, x_i + \sum_{i,j=1}^{M} \sum_{k=1}^{K} w_{ki} w_{kj} x_i x_j$$

where the weight matrix of quadratic terms is a low-rank matrix parameterized by w_{ki}. Given a training set, the FM is trained by minimizing the squared loss. In the reported experiments, the parameter K is set to eight. After the parameters are fixed, quantum annealing is used to identify ten optimal solutions at a time. After that they are added to the training set of the factorization machine. The procedure is repeated 200 times. See the original paper[1] for details.

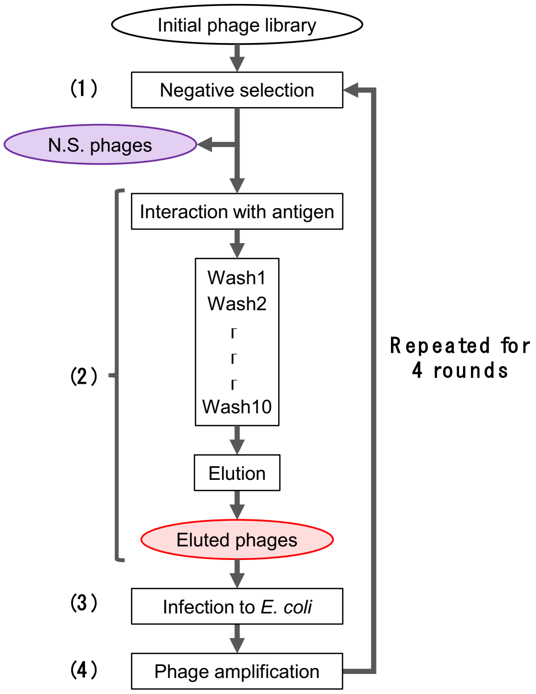

Figure S1 | Workflow of biopanning. In the biopanning, four steps were iterated four times: 1) removal of non-specifically bound phages (negative selection), 2) interaction with antigen and elution of target-binding phages, 3) infection of the selected phage into *E. coli*, and 4) amplification of phages. The phages which were removed at step 1 (purple circle) and which were eluted at step 2 (red circle), are collected for deep sequencing.

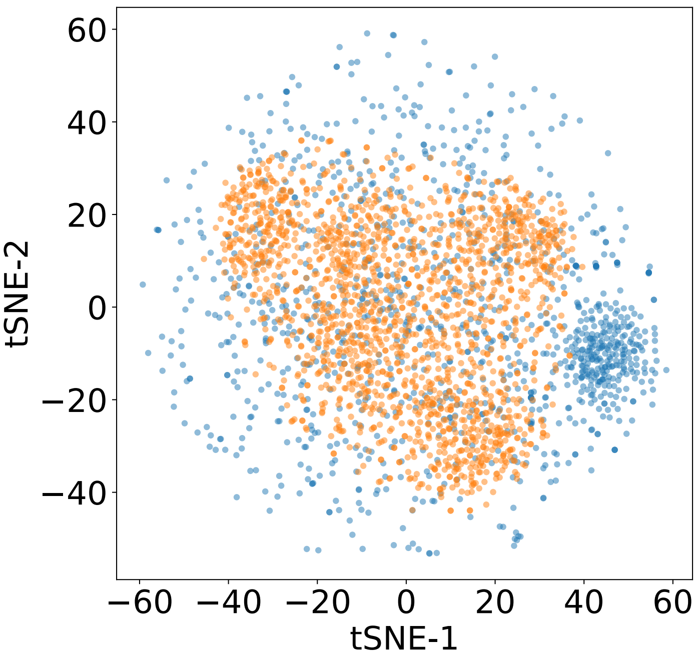

Figure S2 | tSNE representation of training set (blue) and generated sequences (orange).

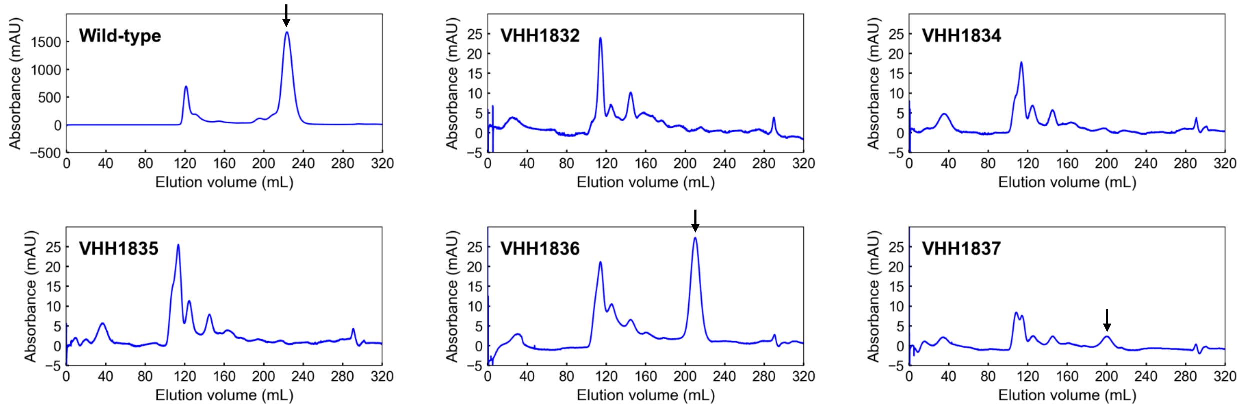

Figure S3 | Size exclusion chromatography. Size exclusion chromatography of the VHHs proposed by MOQA. The wild-type 3DWT and five variants purified by means of immobilized metal ion affinity chromatography were loaded onto a HiLoad 26/600 Superdex 75 pg column. The absorbance of the eluent was monitored at a wavelength of 280 nm. Vertical arrow indicates the fraction of monomeric VHH.

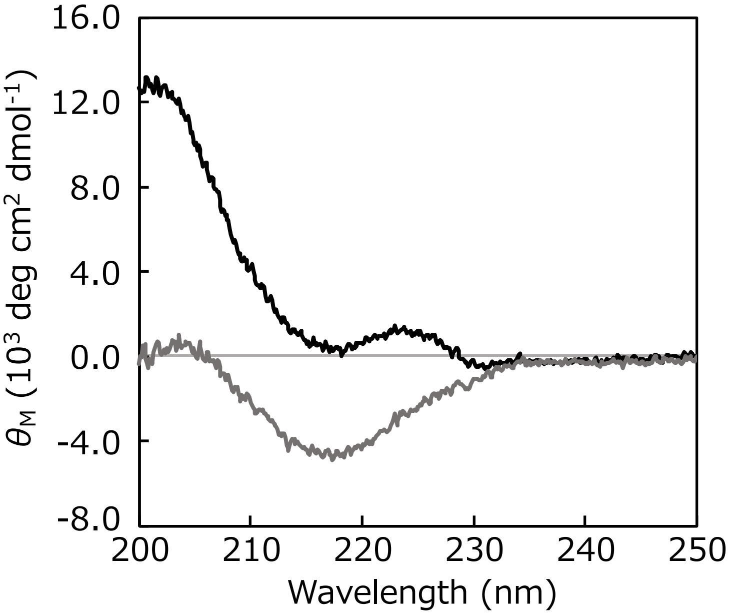

Figure S4 | CD spectra of the wild-type (black) and VHH1836 (gray).

1 Tučs, A. *et al.* Quantum Annealing Designs Nonhemolytic Antimicrobial Peptides in a Discrete Latent Space. *ACS Medicinal Chemistry Letters* 14, 577-582, doi:10.1021/acsmedchemlett.2c00487 (2023).
